# THE INFLUENCE OF STATIC AND DYNAMIC FACTORS ON LONGITUDINAL PATTERNS OF CAREGIVER NETWORK

**DOI:** 10.1093/geroni/igad104.0061

**Published:** 2023-12-21

**Authors:** Jeong Eun Lee, Joseph Svec, Nicholas Cone

**Affiliations:** Iowa State University, Ames, Iowa, United States; Saint Joseph’s University, New York City, New York, United States; Iowa State University, Ames, Iowa, United States

## Abstract

Care networks are defined as the collection of individuals who provide support for older adults with health challenges. Although many older adults receive care from multiple caregivers, with varying intensities of caregiving, little is known about the structure of their care networks and whether or to what extent it changes over time. This study aims to examine the stability of care networks and the predictive effects of static and dynamic factors on patterns of caregiving networks. Using data from the National Health Aging Trends (NHAT) and National Study of Caregiving (NSOC), we examine the static and dynamic intrapersonal structures of care arrangements with regard to systematic continuity across 7 years. We utilize both Latent Profile Analysis and Latent Transition Analysis to identify different types of caregiving arrangements and patterns of transitions over time. Moreover, we use logistic regressions to examine how individual and time-varying factors affect those arrangements and transitions. The results show three groups of caregiving arrangements: 1) intense care with one caregiver, 2) moderate-level care with multiple caregivers, and 3) low-level care with multiple caregivers. Among the groups, moderate-level care with multiple caregivers is the most stable group. Moreover, static factors, such as gender and race, and dynamic factors, such as psychological resilience of care recipients, significantly impacts the probability of transitioning from low-level care to intense care over time. The findings highlight the utility of Latent Transition Analysis in caregiving networks, suggesting that patterns of caregiving outcomes should be understood as process as well as a condition.

